# The Cancer-Associated Virus Landscape in HIV Patients with Oral Hairy Leukoplakia, Kaposi's Sarcoma, and Non-Hodgkin Lymphoma

**DOI:** 10.1155/2012/634523

**Published:** 2012-08-08

**Authors:** Peter D. Burbelo, Joseph A. Kovacs, Jason Wagner, Ahmad Bayat, Craig S. Rhodes, Yvonne De Souza, John S. Greenspan, Michael J. Iadarola

**Affiliations:** ^1^Neurobiology and Pain Therapeutics Section, Laboratory of Sensory Biology, National Institute of Dental and Craniofacial Research, Bethesda, MD 20893, USA; ^2^Critical Care Medicine Department, Clinical Center, Bethesda, MD 20892, USA; ^3^Laboratory of Cell and Developmental Biology, National Institute of Dental and Craniofacial Research, Bethesda, MD 20892, USA; ^4^Department of Orofacial Sciences, School of Dentistry, University of California, San Francisco, San Francisco, CA 94143, USA

## Abstract

Although HIV-positive patients are at higher risk for developing a variety of infection-related cancers, the prevalence of infections with the seven known cancer-associated viruses has not been studied. Luciferase immunoprecipitation systems were used to evaluate antiviral antibodies in four 23-person groups: healthy blood donors and HIV-infected patients with oral hairy leukoplakia (OLP), Kaposi's sarcoma (KS), or non-Hodgkin lymphoma (NHL). Antibody profiling revealed that all HIV-positive individuals were strongly seropositive for anti-gp41 and antireverse transcriptase antibodies. However, anti-p24 HIV antibody levels were highly variable and some OLP and KS patients demonstrated weak or negative responses. Profiling two EBV antigens revealed no statistical difference in antibody levels among the three HIV-infected groups. A high frequency of KSHV infection was detected in HIV patients including 100% of KS, 78% of OLP, and 57% of NHL patients. Most HIV-infected subjects (84%) showed anti-HBV core antibodies, but only a few showed antibodies against HCV. MCV seropositivity was also common (94%) in the HIV-infected individuals and KS patients showed statistically higher antibody levels compared to the OLP and NHL patients. Overall, 68% of the HIV-infected patients showed seropositivity with at least four cancer-associated viruses. Antibody profiles against these and other infectious agents could be useful for enhancing the clinical management of HIV patients.

## 1. Introduction

It is estimated that approximately 18% of all human cancers are caused by infectious agents [1]. A bulk of these cancers are caused by the seven known cancer-associated viruses including Epstein-Barr virus (EBV), hepatitis B virus (HBV), human T-lymphotropic virus-I (HTLV-I), human papilloma virus (HPV), hepatitis C virus (HCV), Kaposi's sarcoma herpesvirus (KSHV; also known as HHV-8), and Merkel cell polyomavirus (MCV) [2]. Although HIV is not a cancer-causing virus, HIV-infected individuals are particularly vulnerable for developing several infection-related malignancies compared to the general population [3–6]. Mechanistically, the increase in malignancy seen in AIDS patients is due to HIV-associated immune suppression and the higher rates of infection by several cancer-associated viruses. In particular, HIV-infected individuals show a high incidence of three AIDS-defining malignancies including KSHV-associated Kaposi sarcoma (KS), HPV-driven invasive cervical cancer, and EBV-associated and nonassociated non-Hodgkin lymphoma (NHL). For KS and NHL, there is a 310-fold and 113-fold higher likelihood, respectively, of developing these malignancies in HIV-infected individuals compared to the general population [4]. There are also other malignancies that are considered AIDS associated including anal cancer, lung cancer, testicular germ cell tumors, and Hodgkin disease, which are more common in HIV than in the general population, but the causative agents are less well defined [6, 7]. 

New tools are needed for identifying individuals who are at risk of developing cancer-virus-associated malignancies, particularly in HIV-infected populations. In general, antibody-based detection of a virus has an advantage over other methods because it can detect both current and previous infections [8]. Antibody-based detection is also especially critical for the diagnosis of many viruses where nucleic acid amplification is not sensitive enough to detect the low levels of viral nucleic acids in plasma after initial infection. For five of the cancer-associated viruses, EBV, HBV, HCV, HTLV-1, and KSHV, the detection of the corresponding antibodies against these agents is only useful for diagnosis of infection and cannot necessarily be used as a biomarker of malignancy. However, the detection of antibodies against certain viral proteins can be specific markers for the presence of the corresponding cancers. For example, anti-E6 and anti-E7 HPV antibodies and anti-T antigen MCV antibodies are often only observed in patients with HPV-driven cancers [9, 10] and Merkel cell carcinoma [11], respectively. Despite these and other findings, the spectrum of coinfection by the seven cancer-causing viruses and the corresponding antibody levels has not been studied in HIV-infected or other human populations.

We have developed the luciferase immunoprecipitation system (LIPS) as a facile platform to quantitatively measure antibodies against a diverse spectrum of infectious agents [8]. LIPS detects robust antibody responses over a wide dynamic range and has been useful for the diagnosis of over 15 different infectious agents including various fungal, bacterial, filarial, and viral pathogens. In addition to using LIPS for highly useful infectious disease diagnostics, LIPS-based antibody profiles can distinguish distinct conditions caused by single infectious agents including HTLV [12, 13], KSHV [14], EBV [15], and HIV [16]. For example, LIPS profiling of the EBV antigens showed much higher antibody levels in chronic active EBV patients compared to healthy blood donors [15]. Similarly, antibody profiling of lytic and latent KSHV antigens distinguished patients with multicentric Castleman's disease from Kaposi sarcoma [14]. Because of these advantages for studying single infectious agents, LIPS is a promising technology for developing comprehensive antibody profiles against multiple infectious agents. Here, LIPS was used to explore, in parallel, the infection status and antibody levels against all seven cancer-associated viruses in HIV-uninfected individuals and HIV-infected patients with OLP, KS, and NHL.

## 2. Material and Methods

### 2.1. Study Patient Samples

Informed written consent was obtained from all subjects in accordance with the human experimentation guidelines of the Department of Health and Human Services under multiple IRB-approved protocols, and the studies were conducted according to the principles expressed in the Declaration of Helsinki. Serum samples (*n* = 23) for the oral hairy leukoplakia (OLP) patients were from the University of California at San Francisco. Both the KS (*n* = 23) and the NHL patient (*n* = 23) samples were from the NIH Clinical Center, NIH. The NHL samples were obtained before therapy. All of the OLP and KS patient samples were taken before 1996, prior to the availability of HAART (nucleoside analog reverse transcriptase inhibitors were available). Additional healthy blood donor controls (*n* = 23) were also used.

### 2.2. *Ruc*-Antigen Fusions for LIPS Analysis of Antibodies against Infectious Agents

The Renilla luciferase (*Ruc*) constructs for influenza [17], HIV-1 [17, 18], EBV [15, 17], HBV [18], HCV [17–20], HTLV-I [12], and KSHV [14, 21] have been described along with their diagnostic performance by LIPS. Additional LIPS diagnostic tests were developed for HPV-16 and MCV. For analyzing antibodies against HPV-16, the E6 and E7 coding sequences were fused to the C-terminus of *Ruc* using the previously described pREN2 vector [22]. For serological studies detecting antibodies against MCV, the VP1 and small T antigens were fused to the C-terminus of *Ruc*. The DNA templates for these two MCV genes were kindly provided by Dr. Christopher Buck (NCI, NIH). DNA sequencing was used to confirm the integrity of these four new antigen constructs. The exact amino acid sequences for these newly described antigens and the PCR primers used to generate each construct are available on request.

### 2.3. LIPS Testing

The general methodology for performing LIPS in a 96-well plate format at room temperature is detailed in a publication and corresponding video [23]. For reiterative antibody profiling by LIPS, a deep-well master plate of serum from HIV-positive and control blood donors was first constructed by diluting serum 1 : 10in assay bufferA (20 mM Tris, pH 7.5, 150 mM NaCl, 5 mM MgCl_2_, 1% Triton X-100) in a 96-well plate. For evaluating antibody titers, 10 *μ*L aliquots of serum (equivalent to 1 *μ*L of serum) from the master plate was added to a polypropylene plate along with 40 *μ*L of bufferA and 50 *μ*L of each *Ruc*-antigen Cos1 cell extract. After 1-hour incubation at room temperature, the IgG antibody-antigen complexes were then captured using a microtiter filter plate containing protein A/G beads for an additional hour. Following washing to remove unbound *Ruc*-antigens, the light units (LUs) were measured by the addition of coelenterazine. All LU data were obtained from the average of at least two separate experiments and not corrected for background protein A/G bead binding. The cut-off values for seropositivity for each antigen were based on values determined from previous studies. However, for determining the infection status of the ubiquitous viral agents, EBV and MCV, the values greater than the mean plus 5 standard deviations of the buffer blanks were used as previously described [17].

### 2.4. Statistical and Data Analysis

The GraphPad Prism software (San Diego, CA) was used for antibody titer data analyses. The nonparametric Mann-Whitney *U* statistical test was used for comparison of antibody titers in different groups. Antibody levels, expressed as mean  log⁡_10_ LU and 95% confidence intervals (CIs), were calculated and presented as antilog values. A heat map was used to visualize individual antibody portraits highlighting the spectrum of infection with different agents and breadth of titers. Antibody levels for each infected sample were calculated as a *Z*-score-based value compared to the uninfected control blood donors or buffer blank. For the heatmap, the order of antibody profiles from left to right consisted of antibody responses against HIV and then on the overall prevalence of the antibodies against the different viruses (EBV, MCV, HBV, KSHV, HCV, and HPV). None of the samples were immunoreactive with HTLV-I and it was omitted from the heatmap. Only the two most informative KSHV antigens are shown. Patient profiles in each group were also manually curated to highlight overall blocks of immunoreactivity against particular cancer-associated viruses.

## 3. Results and Discussion

### 3.1. Defining HIV Immunoreactivity in OLP, KS, and NHL

**Figure 1:**
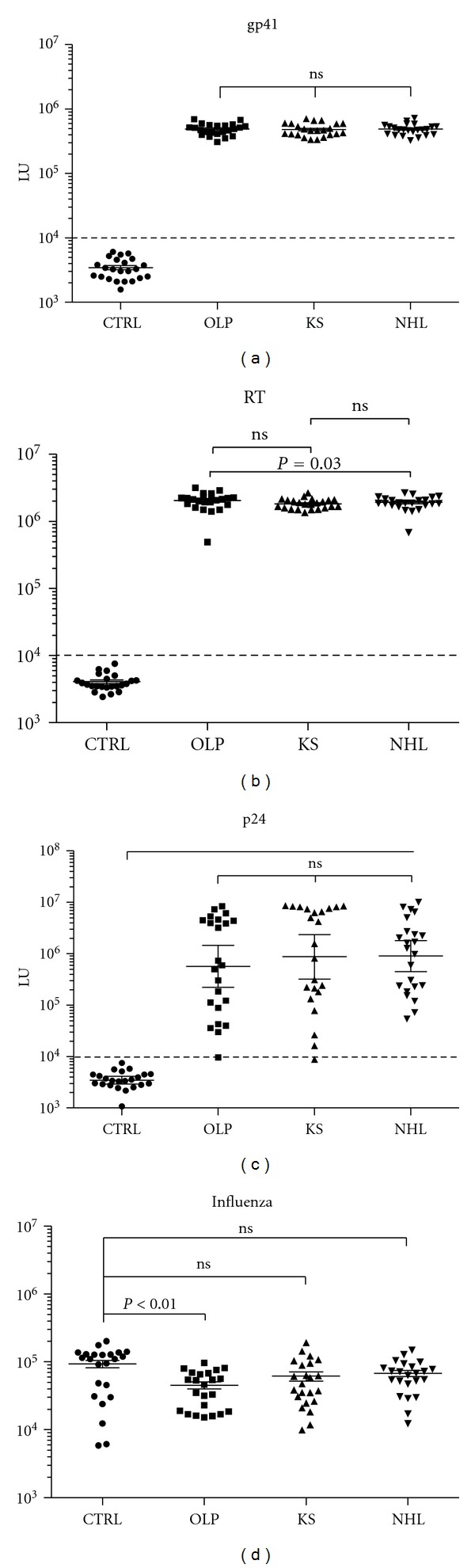
HIV antibody profiling in the uninfected blood donors and HIV patients with OLP, KS, and NHL. The antibody level and 95% CI for (a) gp41 (b) RT, (c) p24, and (d) HA2 influenza antibodies in the 23 control blood donors, 23 OLP, 23 KS, and 23 NHL subjects were plotted on the *Y*-axis using a log⁡_10_ scale. Each symbol represents a sample from one individual. The dashed line represents the cut-off level for determining seropositivity and is derived from the mean plus 5 standard deviations of the antibody values of the controls. *P* values for the different groups were calculated using the Mann-Whitney *U* test.

Antibody titers and infection status were analyzed in two HIV-positive groups with cancer, KS (*n* = 23) and NHL (*n* = 23), as well as a third HIV-positive group with a nonmalignant condition of HIV-associated OLP (*n* = 23). Healthy blood donors (*n* = 23), who were HIV-negative, were also used as controls. Antibodies against the three major HIV antigens, gp41, reverse transcriptase (RT), and p24, were initially evaluated in the four subject groups. While none of the control, uninfected blood donor samples were seropositive against gp41 or RT, all HIV-positive samples were seropositive with similar antibody levels (Figures 1(a) and 1(b)). For example, the levels of antibodies against gp41 in the OLP, KS, and NHL groups were 480,373 (95% CI; 439,507–525,038), 469,728 (95% CI; 425,805–518,182), and 482,215 LU (95% CI; 443,108–524,773), respectively, and by Mann Whitney *U *test no statistical difference in anti-gp41 antibody levels were detected among the three HIV-infected groups (Figure 1(a)). Unlike the gp41 and RT, anti-p24 antibodies demonstrated significant variability in the HIV-infected individuals ranging from 8,676 to 10,600,000 LU (Figure 1(c)). While anti-p24 antibody responses were not statistically different between the OLP, KS, and NHL groups, many of the OLP and KS patients showed blunted anti-p24 antibody responses. One OLP patient and one KS patient were seronegative for p24 antibodies (Figure 1(c)). Additional profiling against the HA2 protein of influenza revealed that all three HIV groups showed antibody levels that were lower than the healthy controls (Figure 1(d)). However, only the OLP group showed statistically (*P* < 0.01) lower influenza antibody levels than the healthy blood donor controls.

### 3.2. Anti-EBV Antibody Profiles

**Figure 2:**
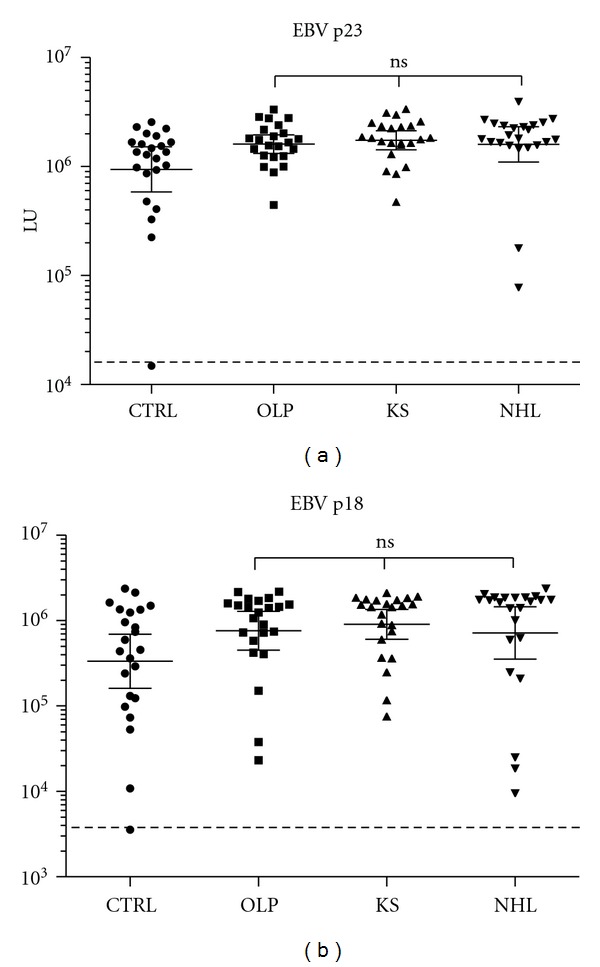
Anti-p23 and p18 antibodies in the uninfected blood donors and HIV-infected subjects. The geometric mean titer and 95% CI for (a) p23 and (b) p18 EBV antibodies in the 23 control blood donors, 23 OLP, 23 KS, and 23 NHL subjects were plotted on the *Y*-axis using a log_10_ scale. Each symbol represents a sample from one individual. The dashed line represents the cut-off level for determining seropositivity. *P* values for the different groups were calculated using the Mann Whitney *U* test.

Previous LIPS studies profiling antibody responses against a panel of EBV antigens demonstrated much higher antibody levels to lytic antigens in chronic EBV patients with high levels of viremia compared to healthy controls [15]. In light of these findings, antibodies against the two major lytic antigens, p18 and p23, were evaluated in this study. As shown in Figure 2, both the anti-p23 and anti-p18 antibody levels were significantly higher (*P* < 0.05) in all three HIV-infected patient groups compared to the uninfected blood donor controls. However, there were no significant differences in anti-p18 or anti-p23 antibody levels among the OLP, KS, and NHL groups (Figure 2). Additional serological testing of 16 different EBV antigens with the same NHL-HIV patient samples failed to detect any significant differences in EBV antibody levels compared to the HIV-positive patients without NHL (data not shown).

### 3.3. Seroprevalence of KSHV Infection

**Figure 3:**
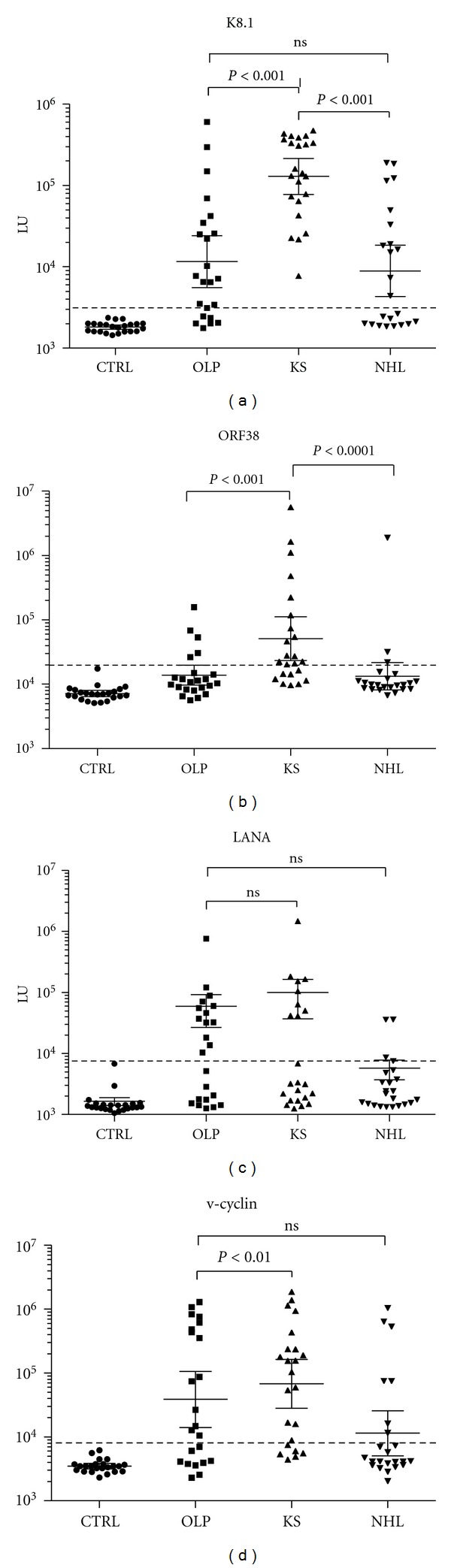
Profiling anti-KSHV antibodies. The antibody levels and 95% CI for (a) K8. 1, (b) ORF38, (c) LANA, and (d) v-cyclin antibodies, in the 23 control blood donors, 23 OLP, 23 KS, and 23 NHL subjects were plotted on the *Y*-axis using a log_10_ scale. Each symbol represents a sample from one individual. The dashed line represents the cut-off level for determining seropositivity and is derived from the mean plus five standard deviations of the antibody values of the controls. *P* values for the different groups were calculated using the Mann Whitney *U* test.

To determine the frequency of KSHV infection and ensure high sensitivity of detection, humoral responses were evaluated by LIPS against two different lytic antigens and two latent KSHV antigens [14, 21]. Using a previously defined cut-off, none of the 23 blood donors were positive for KSHV antibodies (Figure 3). However, all 23 KS patients showed KSHV seropositivity. Examination of the serological responses against the four KSHV antigen panel also revealed that 78% (18/23) of the OLP and 56% (13/23) of the NHL patients were seropositive for KSHV infection. The KS patient group showed the highest antibody levels and spectrum of immunoreactivity against the KSHV antigens (Figure 3). In particular, the KS patients had statistically higher antibody levels (*P* < 0.05) against the K8.1, ORF38, and v-cyclin antigens compared to the OLP and NHL subgroups.

### 3.4. Seroprevalence of HTLV-I, HCV, and HBV Infection

**Figure 4:**
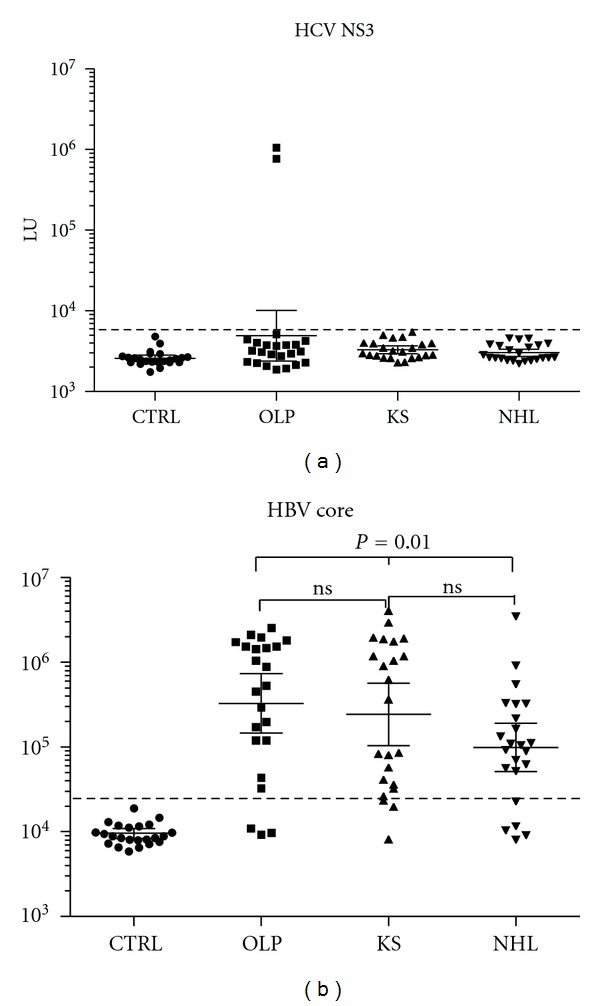
Anti-HCV and anti-HBV antibodies. (a) Antibody profiling against NS3 of HCV was plotted on the *Y*-axis using a log⁡_10_ scale. Only two patients were above the cut-off and HCV seropositive. (b) The anti-HBV antibody levels and 95% CI in the 23 control blood donors, 23 OLP, 23 KS, and 23 NHL subjects were plotted on the *Y*-axis using a log⁡_10_ scale. The dotted line represents the cut-off value.

LIPS has been used previously for the successful serological diagnosis of HTLV-I [12], HCV [17–19], and HBV infection [18]. From evaluating anti-GAG HTLV-I antibodies in the four groups, none of the HIV-positive or HIV-negative subjects were seropositive (data not shown). However, testing for anti-HCV NS3 antibodies in the sample set revealed that two of the 23 OLP patients were HCV seropositive, while none of the KS or NHL subjects were seropositive (Figure 4(a)). Additional profiling revealed that the same two OLP individuals were also copositive for HCV core antibodies confirming infection in these two individuals and none of the other samples (data not shown).

Serological profiling against the HBV core protein revealed varied antibody levels in the HIV-infected subjects ranging from 8,025 to 4,060,000 LU. While the level of antibodies against HBV core antigen in the control blood donor group was 9,263 (95% CI; 8194–10,470), the levels of HBV core antibodies in OLP, KS, and NHL patients were 327,600 (95% CI; 145,700–736,500), 241,800 (95% CI; 103,100–566,900), and 98,970 LU (95% CI; 51,300–190,900), respectively (Figure 4(b)). Based on the defined cut-off value, 87% of the OLP, 87% of the KS, 78% of the NHL, and none of the blood donors were HBV seropositive. Inspection of anti-HBV core antibody levels, omitting seronegative samples from each group, revealed that the NHL patients had the lowest antibody levels among the three HIV groups and the NHL patients had statistically lower levels (*P* = 0.01) than the OLP patients.

### 3.5. Antibody Profiles against MCV and HPV

**Figure 5:**
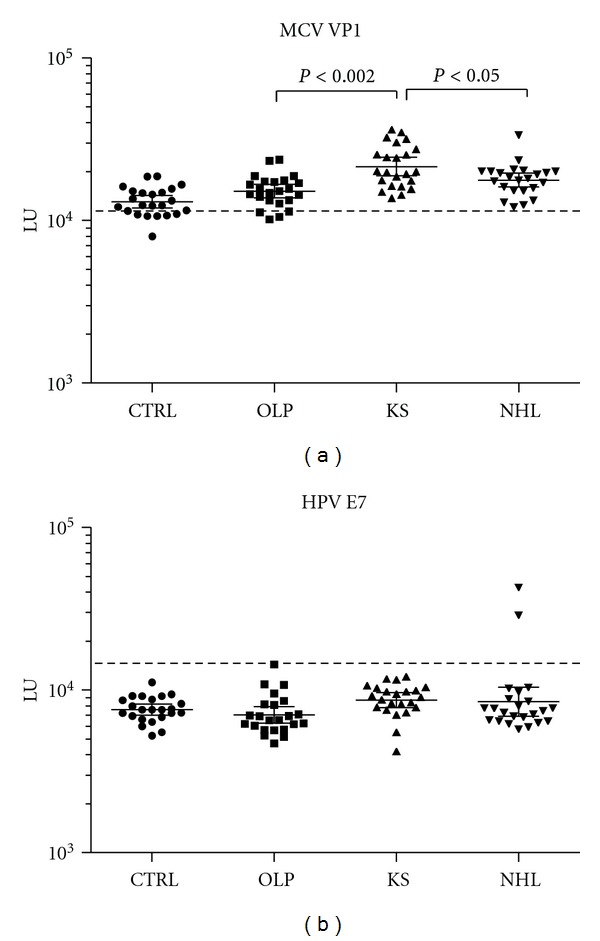
Profiling anti-HPV and anti-MCV antibodies. The antibody levels and 95% CI are shown for (a) MCV VP1 and (b) E7 HPV-16 in the 23 control blood donors, 23 OLP, 23 KS, and 23 NHL subjects. The antibody levels for each individual are plotted on the *Y*-axis using a log⁡_10_ scale. The dashed line represents the cut-off level for determining seropositivity. *P* values for the different groups were calculated using the Mann Whitney *U* test.

Although MCV is a ubiquitous human infectious polyoma virus, MCV-associated Merkel cell carcinoma (MCC), a rare tumor, occurs more frequently in HIV-infected individuals compared to the general population [24]. From LIPS evaluation of MCV VP1 capsid antibodies, a high prevalence of seropositive MCV antibodies was detected in all four subject groups including the control blood donors (Figure 5(a)). Based on the cut-off value, 70% (16/23) of blood donors, 83% (19/23) of OLP, 100% (23/23) of KS, and 100% (23/23) of the NHL were MCV seropositive. Analysis of the anti-VP1 MCV antibodies revealed that all three HIV-infected patient groups showed statistically higher levels than the uninfected blood donor controls. Moreover, the KS patients showed statistically higher anti-VP1 antibody levels (*P* < 0.01) compared to the OLP or NHL patients (Figure 5(a)). In contrast to MCC patients, which often show statistically significant antibodies against the small T antigen of MCV ([11], Burbelo et al., unpublished), no significant humoral responses were detected against the small T antigen in any of the subjects from the four groups (data not shown).

Patients with HPV-driven cervical cancer and oropharyngeal cancer often show serum antibodies against the E6 and E7 viral proteins [9, 10]. LIPS testing against the HPV E7 antigen identified only two seropositive individuals, both of whom were from the NHL subgroup (Figure 5(b)). Additional profiling revealed that these two NHL patients were not positive for anti-E6 HPV antibodies.

### 3.6. Patterns of Infection and Humoral Responses in HIV

**Figure 6:**
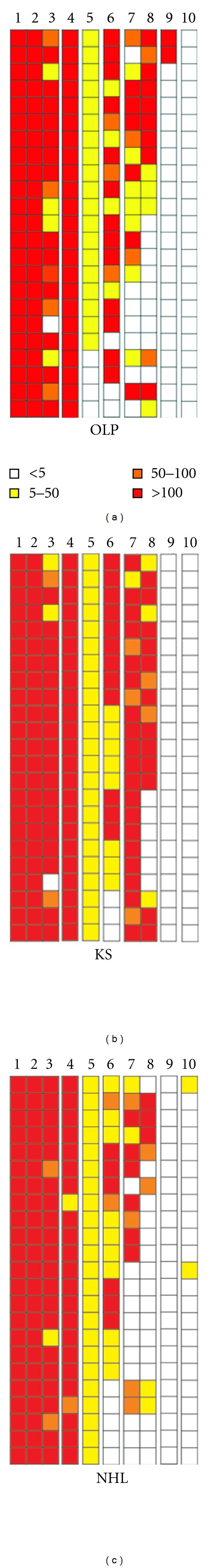
Heatmap analysis of antivirus antibody profiles in HIV patients with OLP, KS, and NHL. Antibody levels were evaluated against (1) gp41 of HIV, (2) RT of HIV, (3) p24 of HIV, (4) p23 of EBV, (5) VP1 of MCV, (6) core of HBV, (7) K8.1 of KSHV, (8) v-cyclin of KSHV, (9) NS3 of HCV, and (10) E7 of HPV. As shown in the key, the color code reflects the relative titers in standard deviations above the mean plus five SD of the control subjects or buffer blanks. Individual antibody profiles were then manually clustered based on the presence or absence of particular antibodies. Each row represents an individual patient.

In addition to analysis of infection with each individual agent, the patterns of infection by multiple agents were also analyzed in the HIV-positive subjects. Heatmap analysis was used to visualize the infection profile of each HIV-infected subject-revealing several findings (Figure 6). First, 68% of the HIV-positive subjects showed infection/exposure with at least four of the cancer-associated viruses including EBV, MCV, HBV, and KSHV. The KS patients showed the highest frequency of infection with these different cancer-associated viruses, in which 87% of the KS were infected with four or more viruses compared to 65% of the OLP and 52% of the NHL group. Two OLP patients showed immunoreactivity to five different cancer-associated viruses. Second, the presence of blunted anti-p24 antibodies in some HIV-infected individuals did not correlate with the presence of any infection or the relative level of other antibodies.

## 4. Conclusions

In this study, the LIPS technology based on luciferase-tagged antigens was employed to generate quantitative antibody profiles against selected antigens from HIV, influenza, and the seven known cancer-associated viruses. To our knowledge, this is the first study to examine infection status and antibody profiles in parallel against these nine different viruses. HIV-infected individuals were the focus of this study since HIV-positive patients are at higher risk for developing a variety of infection-related cancers. Our results with the three groups of HIV patients revealed that 68% of the HIV samples were also coinfected with at least four of the seven cancer-associated viruses (HBV, KSHV, EBV, and MCV). In the case of HBV, the detected antibodies could also reflect patients who have cleared infection. Nevertheless, the high rate of cancer-associated virus exposure was in sharp contrast to the control blood donors who were only infected with the two, EBV and MCV, ubiquitous cancer-associated viruses. Mechanistically the higher antibody levels against EBV and MCV in the HIV-infected patients compared to the healthy controls likely reflects the loss of immune control over these agents resulting in increased viremia and antibody production. On the other hand, these finding for EBV and MCV were not due to a generalized increase in antibody production in HIV-positive patients because antibody levels against the HA2 antigen of influenza were lower in the HIV patients.

Previous studies have shown that the pathogenesis of OLP involves EBV infection of epithelial cells in the oral cavity [25]. Although it was expected that the anti-EBV antibody levels would be higher in the OLP group compared to the KS group, this was not the case. The antibodies against the EBV p23 and p18 antigens in the OLP group were not statistically different than the levels found in the KS group or NHL groups. It is possible that altered anti-EBV antibody responses in the OLP patients might exist in saliva, but this needs to be studied further. Despite these negative findings, the OLP patients did show two features of having impaired humoral immunity. First, many of the OLP patients showed blunted anti-p24 HIV antibody responses. A few KS patients also showed low levels of anti-p24 HIV antibodies. However, these low levels were not a general defect of humoral immunity because these same OLP and KS patients showed comparably high levels of antibodies against other HIV antigens (i.e., gp41 and RT) and many of these same patients showed robust humoral responses against the HBV core antigen. Additional dilution experiments involving the titration of anti-p24 antibodies in patient serum samples failed to demonstrate that the low titers of anti-p24 antibodies were due to p24 antigen-antibody immune complexes (Burbelo, unpublished). As previously reported, the low anti-p24 antibody responses in the patients are more likely due to T-cell exhaustion [26] and may be a marker of AIDS progression [27]. The OLP group also showed the poorest humoral responses against influenza. These results are consistent with other reports showing that HIV patients with low CD4 counts have depressed immunity against influenza [28, 29]. It is perhaps appropriate to point out that many of the OLP patients were often coinfected with several cancer-associated viruses including 78% with KSHV, 90% with HBV, and 8% with HCV. Although other studies have shown that KSHV infection is frequently found in cohorts of men who have sex with other men (~20–45%) [30, 31] and is highly correlated with HIV infection [30, 32, 33], the high frequency (78%) of KSHV infection in patients with OLP was unexpected. Based on the high prevalence of KSHV infection in the OLP patients and the ability of KSHV to propagate in the oral cavity [34], it is tempting to speculate that the KSHV infection might contribute to the immunosuppression seen in the oral cavity of some OLP patients.

The KS patients showed the highest frequency of infection with these different cancer-associated viruses. Although the KS patients showed the highest levels of anti-KSHV antibodies, this was not a consistent trend and there were selected OLP and NHL patients with equally high antibodies. The KS patients also had the highest antibody levels against MCV VP1 capsid protein compared to the control blood donors, OLP, or NHL patients. Since MCV and KSHV share a tropism for cells in the dermis [2], the higher antibody levels seen against MCV-VP1 in the KS group could reflect an enhanced immune attack on the skin.

EBV has been implicated as contributing to HIV-associated NHL, especially immunoblastic and central nervous systems types. In contrast to several previous studies correlating antibodies with a particular infectious disease condition [12, 14–16], antibody profiles against EBV were not informative biomarkers of NHL. These findings are also consistent with a recent study using a protein array of 40 different EBV proteins, which failed to detect informative NHL-specific EBV humoral responses [35]. This negative data regarding antibody profiling may reflect the pathogenesis of HIV-associated NHL, which predominantly involves a loss of T-cell immunity and genomic rearrangements [36, 37]. Nevertheless, the NHL subgroup did show the lowest anti-HBV core antibody levels among the HIV-infected groups, despite having a similar prevalence of HBV infection. It is possible that the lower anti-HBV core antibodies in the NHL patients is a surrogate of impaired immune function, but this requires further exploration.

It is important to point out that our study has several limitations. First, asymptomatic HIV-positive individuals were not studied to determine the prevalence of infection for these viruses. Second, the HIV individuals used for analysis may not necessarily be representative patients of OLP, KS, or NHL and it is highly likely that the prevalence of many of these viral coinfections may be markedly different in other cohorts of patients. The high rate of HBV infection seen in our cohort is of particular importance because HBV infection has a significant negative impact on HIV outcome [38]. While the HIV patient serum samples studied here were from the 1980–1990s, more recent vaccination and other preventive measures have greatly changed the level of HBV infection in the United States [39, 40]. It is also important to point out that HIV-infected individuals show a low rate of response to vaccination and therefore remain at risk for HBV infection [41]. Similarly, the incidence of KS, and to a lesser extent NHL, has decreased with HAART [42, 43], which may also influence the relative antibody levels seen in those patients. Another limitation is our selection of antigens. Along these lines, antibodies were only examined against two HPV proteins, not against the capsid, but it is likely we underestimated the prevalence of asymptomatic HPV infection in these subjects. Although the long-term followup of the HIV patients is lacking, it is possible that some of these HIV-positive subjects may have gone on to develop clinical problems related to some of these viral coinfections. For example, it is possible that some of the OLP patients were infected with KSHV ultimately when on to develop KS.

Our study shows the feasibility of generating highly quantitative antibody profiles against multiple viruses including HIV, influenza, and seven cancer-associated viruses. The findings of varying anti-viral antibody levels against influenza, KSHV, HBV, and MCV likely involve multiple causes including alterations of specific immune pathways in the HIV-infected individuals that are pathogen-specific, the levels of immunosuppression, and/or the time of viral infection in relation to infection with HIV. Since many of these viruses can cause not only cancer, but other illnesses, serological screening may be helpful for the clinical management of these patients. For example, HBV and HCV infections are also associated with liver disease in HIV-infected individuals, which is also a major cause of morbidity [44] and thus serological screening may have utility for diagnosis and monitoring. The ability to individually profile these cancer-associated viruses, along with many other infectious agents, has the potential to provide high quality humoral response data that could be correlated with other medically relevant inventories and be used as a platform for developing a generalized disease surveillance technology.
